# A New Clevosaurid from the Triassic (Carnian) of Brazil and the Rise of Sphenodontians in Gondwana

**DOI:** 10.1038/s41598-019-48297-9

**Published:** 2019-08-14

**Authors:** Annie S. Hsiou, Randall L. Nydam, Tiago R. Simões, Flávio A. Pretto, Silvio Onary, Agustín G. Martinelli, Alexandre Liparini, Paulo R. Romo de Vivar Martínez, Marina B. Soares, Cesar L. Schultz, Michael W. Caldwell

**Affiliations:** 1 Laboratório de Paleontologia, Universidade de São Paulo, Ribeirão Preto, São Paulo, Brazil; 2 Arizona College of Osteopathic Medicine and Department of Anatomy, College of Graduate Studies, Midwestern University, Glendale, AZ, 85308, USA; 3 Department of Biological Sciences, University of Alberta, Edmonton, Canada; 4 Present Address: Department of Organismic and Evolutionary Biology, Museum of Comparative Zoology, Harvard University, Cambridge, MA 02138 USA; 5 CAPPA - Centro de Apoio à Pesquisa Paleontológica da Quarta Colônia, Universidade Federal de Santa Maria, São João do Polêsine, Brazil; 6 CONICET-Sección Paleontología de Vertebrados, Museo Argentino de Ciencias Naturales ‘Bernardino Rivadavia’, Buenos Aires, Argentina; 7 Laboratório de Paleontologia de Vertebrados, Universidade Federal do Rio Grande do Sul, Porto Alegre, Brazil; 8 PIBi-Lab – Laboratório de Pesquisas Integrativas em Biodiversidade, Departamento de Biologia, Universidade Federal de Sergipe, São Cristóvão, Sergipe Brazil; 9 Department of Earth and Atmospheric Sciences, University of Alberta, Edmonton, Canada

**Keywords:** Taxonomy, Palaeontology, Phylogenetics

## Abstract

The early evolution of lepidosaurs is marked by an extremely scarce fossil record during the Triassic. Importantly, most Triassic lepidosaur specimens are represented by disarticulated individuals from high energy accretion deposits in Laurasia, thus greatly hampering our understanding of the initial stages of lepidosaur evolution. Here, we describe the fragmentary remains of an associated skull and mandible of *Clevosaurus hadroprodon* sp. nov., a new taxon of sphenodontian lepidosaur from the Late Triassic (Carnian; 237–228 Mya) of Brazil. Referral to Sphenodontia is supported by the combined presence of a marginal dentition ankylosed to the apex of the dentary, maxilla, and premaxilla; the presence of ‘secondary bone’ at the bases of the marginal dentition; and a ventrally directed mental process at the symphysis of the dentary. Our phylogenetic analyses recover *Clevosaurus hadroprodon* as a clevosaurid, either in a polytomy with the Late Triassic to Early Jurassic *Clevosaurus* and *Brachyrhinodon* (under Bayesian inference), or nested among different species of *Clevosaurus* (under maximum parsimony). *Clevosaurus hadroprodon* represents the oldest known sphenodontian from Gondwana, and its clevosaurid relationships indicates that these sphenodontians achieved a widespread biogeographic distribution much earlier than previously thought.

## Introduction

The Triassic period marks one of the great transitions in the history of life after the mass-extinction at the end of the Permian. Most notably, this was a period of diversification of several major groups of extant terrestrial and marine vertebrates^1^, including lepidosaurian (Squamata + Rhynchocephalia) reptiles^2^. Today, Lepidosauria is the largest and the most diverse group of non-avian reptiles with over 10,000 known species^3^. Most of these are lizards and snakes with rhynchocephalians limited to two recognized sphenodontian species (*Sphenodon punctatus* and *S*. *guntheri*) in New Zealand^4^. However, during the early Mesozoic, rhynchocephalians (particularly sphenodontians), were the most diverse and widely distributed members of Lepidosauria^5^.

In the last decade, the discovery of several important fossils has greatly increased our knowledge of the anatomy, systematics, distribution, and early evolution of sphenodontians. These new findings include presumably stem rhynchocephalians of the Late Triassic, such as *Gephyrosaurus evansae*^6^ and *Penegephyrosaurus curtiscoppi*^7^ from the United Kingdom, and *Deltadectes elvetica*^7^ from Switzerland. These three taxa, though not being well-understood phylogenetically, have been tentatively positioned as stem rhynchocephalians, sharing morphological features with *Gephyrosaurus* and closely related taxa^6,7^.

By far, the most well-known early sphenodontian clade is Clevosauridae, which currently includes several species allocated to three genera: *Brachyrhinodon*, *Clevosaurus*, and *Polysphenodon* from the Late Triassic to Early Jurassic of Britain^8–12^, Germany^9,13,14^, Africa^15,16^, China^17^, North America^18^, and South America^19^. Although support for this clade has not been universal^9,20–22^, the genus *Clevosaurus* is currently considered the most specious (six species) and widely distributed (Asia, Europe, North America, South America, Africa) of the clevosaurid taxa^17,18,23–25^.

The new sphenodontian taxon described herein is the oldest unequivocal record of Rhynchocephalia (Sphenodontia) from South America, as well as in Gondwana. It provides the first evidence of a greater diversity of early sphenodontians than previously understood for southern Pangaea and provides new information on the earliest stages of the acquisition of the acrodont dentition in sphenodontians in combination with unique dental specializations.

## Results

### Systematic Palaeontology

Lepidosauria Dumeril and Bibron, 1839 *sensu* Evans, 1984

Rhynchocephalia Günther, 1867 *sensu* Gauthier *et al*., 1988

Sphenodontia Williston, 1925 *sensu* Benton, 1985

Referral to Sphenodontia is supported by the combined presence of a marginal dentition ankylosed to the apex of the dentary, maxilla, and premaxilla; the presence of “secondary bone” (*sensu* Harrison^26,27^, Fraser^28^ and Jones^22^) at the bases of the marginal dentition; a ventrally directed mental process (*sensu* Apesteguía^29,30^) at the symphysis of the dentary; presence of a very shallow Meckelian canal.

Clevosauridae Bonaparte and Sues, 2006 (*sensu* this study)

### New definition

Clevosauridae was originally defined by Bonaparte and Sues^19^ as the least inclusive clade containing the last common ancestor shared between *Brachyrhinodon*, *Polysphenodon*, and *Clevosaurus* plus all of its descendants. Due to the uncertain position of *Polysphenodon* (*i*.*e*. if the genus is or is not closely related to the sister group *Brachyrhinodon* + *Clevosaurus*) in other topologies^9,20,21,25^, here we adopt the interpretation of Clevosauridae as a monophyletic group using the node–based definition of the clade containing the last common ancestor shared between *Brachyrhinodon* and *Clevosaurus* plus all of its descendants.

Clevosaurus hadroprodon sp. nov.

### Etymology

Species epithet comes from a combination of ‘hadroprodon’ (from the Greek “hadros”-meaning large) and “protos”, meaning first (Greek), and “odous”, meaning tooth (Greek); in reference to the “big first tooth”, i.e. the large tusk-like tooth of the premaxilla and the anteriormost/first dentary tooth position.

### Holotype

MMACR PV-027-T, an incomplete right premaxilla, an incomplete right maxilla and right lower jaw in occlusion, and left lower jaw located below these elements.

### Referred material

MMACR PV-028-T, an incomplete left lower jaw; and MMACR PV-029-T, fragment of dentary with three isolated teeth.

### Locality and horizon

**Figure 1:**
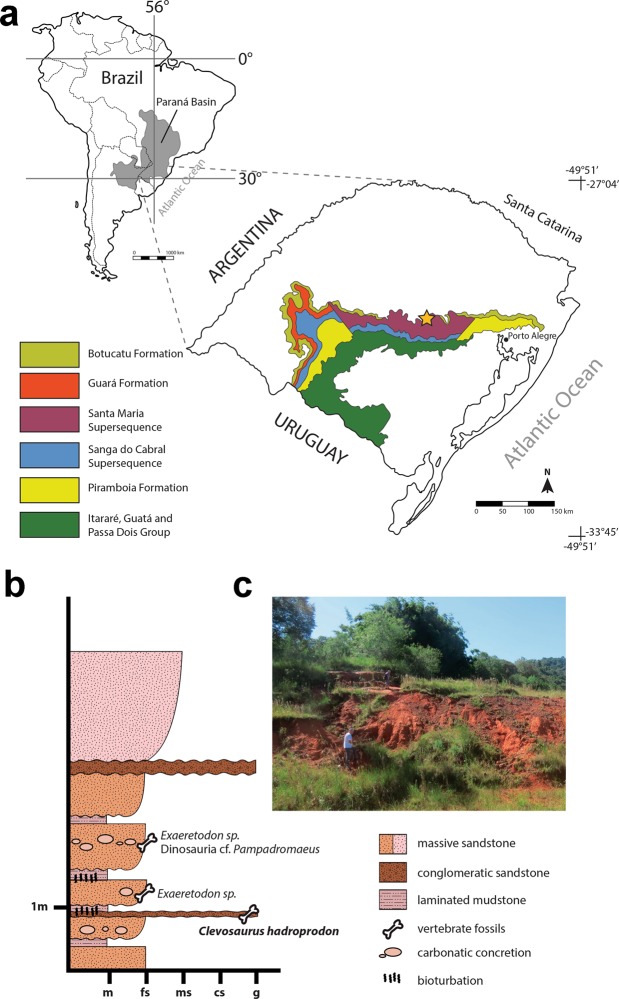
Location and stratigraphy interpretation. Geological map (**a**) of Paraná Basin in South America (upper left) and showing the Santa Maria Supersequence in Rio Grande do Sul state (southern Brazil) (detail). The Sanga do Cabral and Santa Maria Supersequences represent the geographical extent of Triassic strata. The star marks the location of the municipality of Candelária where the specimen here described comes from (Modified from Horn *et al*.^30^). Summarized stratigraphic scheme of the Linha Bernardino outcrop (**b**), type locality of *Clevosaurus hadroprodon* sp. nov. Panoramic view of the Linha Bernardino type locality (**c**).

Linha Bernardino locality, Candelária municipality, Rio Grande do Sul State, Southern Brazil (Fig. 1); Santa Maria Formation (Santa Maria Supersequence, Candelária Sequence), Rosário do Sul Group, Paraná Basin; Carnian, Late Triassic^31,32^ (Fig. 1a–c). *Clevosaurus hadroprodon* was recovered from immediately beneath the layers that contained the cranial and postcranial remains of the cynodonts *Exaeretodon* sp. and *Trucidocynodon* sp. and a distal portion of femur that closely resembles the early dinosaur cf. *Pampadromaeus* (Fig. 1b). The presence of these fossils referred to *Exaeretodon*, *Trucidocynodon* and a *Pampadromaeus*-like form places this locality within the *Hyperodapedon* AZ at the base of the Candelária Sequence. Recent high-precision U-Pb zircon geochronology data recovered a weighted mean ^206^Pb/^238^U date of 233.23 ± 0.73 Ma for typical *Hyperodapedon* AZ sites^33^, which can be biostratigraphically correlated to the sedimentary layers containing *C*. *hadroprodon*. As such we conservatively consider the age for the fossil material to be Carnian (Late Triassic).

### Diagnosis

Small sphenodontian rhynchocephalian differing from all other known sphenodontians in possessing the following combination of features: (1) a large, blunt, tusk-like tooth in both premaxilla and first tooth position of dentary; (2) in having an angled, but nearly vertical mandibular symphysis and relatively deep dentary; (3) absence of an edentulous gap between the tusk-like tooth and the remaining dentition; (4) absence of a posterodorsal process of the premaxilla; (5) in lacking well-developed medial-posteromedial expansion of the posterior dentition; (6) and lacking flanges on the teeth, and (7) lacking labially expanded teeth. Feature 4 makes this new taxon different from many sphenodontians, including other species of *Clevosaurus*, *Rebbanasaurus*, *Godavariasaurus*, *Priosphenodon*, *Sphenotitan*, *Paminzisaurus* and *Ankylosphenodon*. Features 5 and 6 make this taxon different from all other described species of *Clevosaurus* [*Clevosaurus hudsoni*, *C*. *bairdi*, *C*. *latidens*, *C*. *sectumsemper*, *C*. *cambrica*, and *Clevosaurus* sp. (China)]

### Description and taxonomic comparisons

#### Premaxilla

**Figure 2:**
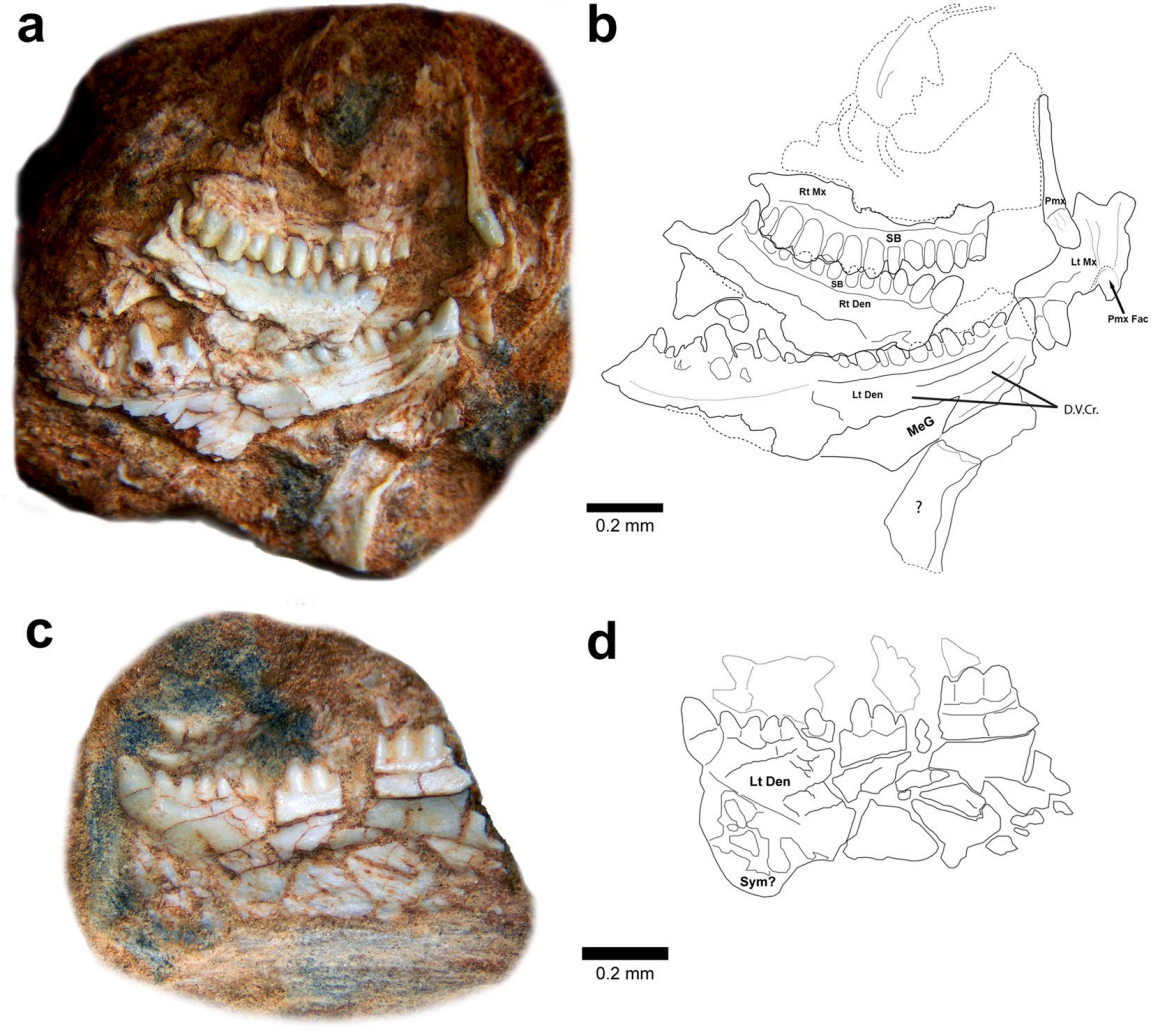
Type and referred specimens of *Clevosaurus hadroprodon* sp. nov. Photograph (**a**) and outline (**b**) of holotype partial skull (MMACR PV-027-T). Photograph (**c**) and outline (**d**) of referred partial left dentary (MMACR PV-028-T). Photograph (**e**) and outline (**f**) of posterior view of section in box in c showing cross sectional view of tooth attachement. Abbreviations: Lt Den, left dentary; Lt Mx, left maxilla; MeG, Meckel’s groove; Pmx, right premaxilla; Pmx Fac, premaxillary facet in maxilla; Rt Den, right dentary; Rt Mx, right maxilla; SB, secondary bone; D.V.Cr., dentary ventral crest; Sym, symphyseal region of dentary.

The right premaxilla of the holotype MMACR PV-027-T (Fig. 2a,b) appears to be complete, but is not articulated with the maxilla and is exposed in lingual/posterior view. It bears a single, massive, tusk-like tooth and has a narrow, long (almost 3 mm), mediolaterally compressed, straight ascending nasal process with a slight (approximately 20°) medial deflection with respect to the long axis of the tooth. Because the anterior portion is embedded in the matrix the exact anteroposterior depth of this process is unknown, but it was clearly greater than the mediolateral width. There is no evidence of a palatal portion, incisive process, expanded maxillary process, or a second ascending maxillary process. It appears that the inferior portion of the premaxilla consists only of the bone necessary for attachment of the single tooth.

The nasal process of the premaxilla in *Clevosaurus hadroprodon* differs from the one in *Diphydontosaurus*^34^, *Gephyrosaurus*^35^, and *Planocephalosaurus*^36^ by being vertically-oriented rather than curved. Additionally, the premaxillae of the latter three taxa are more squamate-like than sphenodontian-like in possessing several small teeth along a laterally expanded alveolar portion of the bone. Additionally, *C*. *hadroprodon* does not show the wide palatal shelf on the medial portion of the premaxilla as seen in *Gephyrosaurus*^35^ and *Diphydontosaurus*^34^. A nearly vertical nasal process of the premaxilla is also present in *Rebbanasaurus*, *Godavariasaurus*^21^, and other species of *Clevosaurus*^28^. In *Rebbanasaurus* and *Godavariasaurus* the process is shorter than that of *C*. *hadroprodon*. In most *Clevosaurus* species the nasal processes tend to be long, with the condition in *C*. *brasiliensis*^19^ being the most similar to *C*. *hadroprodon* in relative length. However, differing from *C*. *hadroprodon*, *C*. *brasiliensis* also possesses a posterodorsal process of the premaxilla^19,21,28^, which is typical of most other known sphenodontians (e.g., *Homoeosaurus*, *Palaeopleurosaurus*, *Piocronus*, *Pleurosaurus*, *Priosphenodon*) and is also present in many archosauromorphs as well as the lepidosauromorphan *Kuehneosaurus*^22^. The absence of this process evidenced that only maxilla could have formed the posterior margin of the external naris. This feature is also seen in the early rhynchocephalians, *Gephyrosaurus*^35^ and *Diphydontosaurus*^34^, respectively, as well as in *Sphenodon*.

A single premaxillary tooth is also present in the *Clevosaurus bairdi*^18^, *C*. *brasiliensis*^25^ as well as in *Kallimodon* (pers. obs. TRS) and *Sphenodon*. The single premaxillary tooth of *C*. *hadroprodon* is massive and cylindrical making it more characteristically tusk-like. The longitudinal groove on the premaxillary tooth is likely a shallow facet on the distal surface (terminology following Smith and Dodson^37^) of the tooth to accommodate the tip of the corresponding tusk-like tooth in the first dentary tooth position.

#### Maxilla

The right maxilla of the holotype (MMACR PV-027-T) is exposed in lateral view (Fig. 2a,b), preserving the portion directly associated with the tooth row, but is otherwise badly crushed and missing the articular portions of the anterior and posterior margins. The preserved portion is mostly the robust, smooth “secondary bone” (see below) that forms at the bases of the teeth which is typical of many sphenodontians. A small portion of the facial process is exposed posteriorly and appears to form a curved posterior process rising above the terminal tooth. This may be the jugal process but is too poorly preserved to be certain. The left maxilla is embedded in the matrix deep to the other elements, but the anteriormost portion is visible in medial view inferior to the premaxilla. Anterior to the two preserved teeth (rotated slightly posteriorly due to breakage of the jaw) is what appears to be a short “diastema”. Anterior to this is a deeper notch, presumably the articulation facet for the premaxilla. Although poorly preserved, the anterior margin of the facial process appears similar to that of most *Clevosaurus* species in being tall, vertical, and ascending from above the anterior margin of the premaxillary facet. The maxillary dentition is similar to that of the dentary (see below).

#### Dentary

The anterior portions of both the left (medial view) and right (lateral view) dentaries are present in MMACR PV-027-T (Fig. 2a,b) and the lateral view of the anterior portion of the left dentary is preserved in MMACR PV-028-T (Fig. 2c,d). All three of the dentary specimens are crushed making it difficult to evaluate the anatomy inferior and posterior to the more robust anterior alveolar portions. None of the lower jaw specimens preserve discernable postdentary elements. Based on the preserved portion (nearly 10 mm) of the tooth row in the left dentary of the holotype, this element is relatively long and deep. The best-preserved portion of both dentaries is the massive subdental bone associated with the anterior portion of the tooth row. On the holotype dentaries this subdental bone is exposed in both lateral and medial views. The lateral side is convex, and bears a distinct, ventrally directed, mental process (particularly apparent on MMACR PV-028-T, Fig. 2c,d). There appear to be several small mental foramina near the symphysis (holotype MMACR PV-027-T, Fig. 2a,b), but the surface is heavily pitted, and it is not possible to confidently distinguish the pittings from possible foramina. The medial surface is more vertical with a tall, shallow longitudinal groove extending from the base of the anteriormost tooth to approximately the eighth tooth position. The anteriormost end of this groove appears to extend to the symphysis, but it is not clear if this is not an artifact of the damage to the symphysial portion of the element. More posteriorly the subdental bone flattens, appears blade-like, and bears an articulation facet presumably for the squamous contact with the articular and possibly the prearticular. Inferior to the subdental bone is the deep Meckel’s groove, which is widely open at the symphysis, narrows posteriorly as a short isthmus, and then expands again as it continues posteriorly. The inferior and posterior portions of the dentary are not sufficiently well preserved to further evaluate the Meckel’s groove. The symphyseal portion of the dentary was formed from a relatively thin, ventrally oriented tab that projected below the inferior margin of the dentary.

In comparison with other sphenodontians, the dentary of *Clevosaurus hadroprodon* is unique in its overall structure. It is apparently similar in its substantial depth but with a much longer tooth row than those of adult *C*. *brasiliensis* (see Romo de Vivar and Soares^38^ for a review of the ontogeny of *C*. *brasiliensis* mandibles). Many sphenodontians with relatively long tooth rows such as living *Sphenodon* and such fossil taxa as *Sphenocondor*, *Sphenovipera*, and *Tingitana*^29,39,40^ also have mandibles that are long and gracile. Most crown-group sphenodontians are characterized by having dentaries that are relatively short and deep.

The shallow groove dorsal to the Meckelian canal near the symphysis is similar to that described for *Cynosphenodon*^41^, but differs in *Clevosaurus hadroprodon* in being longer and possessing a narrow, rather than deep, ridge of bone separating it from the Meckelian canal. If the narrow extension of this groove beneath the base of the tusk-like dentary tooth towards the symphysis is not an artifact of preservation, then, as with *Cynosphenodon*^41^, it may indicate that the holotype specimen of *C*. *hadroprodon* is a juvenile (probably between the T3-T4 stages of Robinson^42^).

The Meckelian canal of *Clevosaurus hadroprodon* is most similar to that found in *C*. *brasiliensis* (Hsiou *et al*.^25^) and *Sphenotitan leyesi*^43^ in being widely open with a constriction just posterior to the symphysis. A dentary (NHMUK R6102) referred to *Clevosaurus hudsoni* also has a widely open Meckelian canal, but lacks a conspicuous isthmus posterior to the symphysis, but another dentary (UMZC T1307) for the same taxon does exhibit this morphology suggesting it may be variable (TRS, pers. obs.). The groove dorsal to the Meckelian canal in *C*. *hadroprodon* is more pronounced than the narrow groove found in one of the dentaries of *C*. *hudsoni* (UMZC T1307), but in the case of *C*. *hadroprodon*. the dorsal margin of the groove is bounded by bone rather than by the ventral edge of “secondary bone”.

#### Dentition

The holotype has heterodont dentition in the upper and lower marginal tooth rows characterized by the distinct dental regions developed in mesiodistal (anteroposterior) sequence (Fig. 2a–d). This includes one anteriormost successional tooth, represented by the single, large, tusk-like tooth on the dentary (or ‘caniniform’ successional tooth, see Apesteguía *et al*.^29^), and an equivalent tusk-like tooth in the premaxilla. *Clevosaurus hadroprodon* has an alternating tooth series distal to the first tooth position in the dentary represented by at least three teeth. There could be additional dentary teeth in this series, but these are not observable in both dentaries due to overlapping of the jaw elements. The anterior portion of the maxilla also has an alternating tooth series, represented by the first six preserved maxillary teeth in the right maxilla. Finally, the alternating series in both jaws is succeeded distally by an additional tooth series, comprised of six preserved teeth in the right maxilla. The number of teeth in the additional series on the dentaries is unknown due to poor preservation of the posterior dentary region.

The single successional tooth of the premaxilla is massive, conical, blunt (tip may be worn), and vertically oriented. The base is firmly ankylosed to the premaxilla and there is no evidence of “secondary bone” (*sensu* Harrison^26,27^; Jones^22^; AKA “secondary dentine” of Fraser^28^; since the histological characteristics of this tissue have not yet been demonstrated we have adopted the more inclusive term ‘secondary bone’ but use quotes to denote uncertainty of its specific structure, but see Jones^22^ for a review of alternative terminology).

The tusk-like tooth in the dentary occupies the first tooth position above the symphysis. This tooth, similar to that of the premaxilla, is mesiodistally massive, conical in shape, and apicobasally procumbent (approximately 30°), and is attached to the tip of the dentary slightly below the attachment region of the remaining dentary teeth.

The remaining teeth of the dentaries and the maxillae are similar in morphology. The longest of the dentary tooth rows preserves approximately 19 tooth positions and the most complete maxillary tooth row has 12 tooth positions, though both are broken posteriorly suggesting that several teeth are missing in both the upper and lower marginal tooth rows. Apart from the tusk-like teeth in the first dentary tooth position and in the premaxilla, all the preserved tooth crowns are similar in being labiolingually compressed, generally triangular, and with distinct mesiodistal carinae, but lacking the labiolingual flanges common to other sphenodontians. The teeth also differ from each other in relative size along the tooth row with shorter, narrower (mesiodistally) teeth anteriorly in the dentary and maxilla and taller, wider teeth posteriorly, but with smaller teeth interspersed among these larger teeth. The exact pattern cannot be determined due to damage along each of the tooth rows. There is no evidence in any of the specimens of active tooth replacement.

In the dentaries there is a thin ridge of “secondary bone” that obscures the tooth-jaw contact in both labial and lingual views with the labial being more prominent. All tooth bases of the holotype are fused and ankylosed to the apex of the jaws and lack any discernable evidence of active tooth replacement. There is no clear evidence of this tissue at the bases of the maxillary teeth, but only the labial surface is clearly visible. The “secondary bone” lacks any evidence of wear facets.

### Comparative osteology of *Clevosaurus hadroprodon* to other rhynchocephalians

*Clevosaurus hadroprodon* can be classified within the Rhynchocephalia, and more specifically within Sphenodontia, based on several characters of jaw morphology: both anterior and posterior tooth series apically placed and ankylosed to the jaw bone labial margin; presence of ‘secondary bone’ deposition along the tooth-jaw contact; a dorsoventrally deep and vertically oriented symphyseal margin of the dentary; deep ventral crest of the dentary medial wall; and the presence of an anterior canine tooth that is apically placed and fused to the dentary. Although some of these features also occur within acrodontan squamates (which generally have a remarkable degree of convergence with sphenodontians), the combination of features in *C*. *hadroprodon* is only seen in sphenodontians (see Supplementary Data 1).

*Clevosaurus hadroprodon* shares with most rhynchocephalians the presence of acrodont tooth implantation in association with ‘secondary bone’. This does differ from the early rhynchocephalians *Gephyrosaurus* and *Diphydontosaurus* in that these two taxa lack ‘secondary bone’ and are characterized by pleurodont attachment of polyphyodont teeth along the entire tooth row (*Gephyrosaurus*), or, the combination of anteriorly pleurodont and posteriorly acrodont attachment (*Diphydontosaurus*)^21,28,34,35,44^. *Clevosaurus hadroprodon* is thus more similar to later evolving forms by having a fully acrodont tooth implantation, and most of them also develop the ‘secondary bone’ in mature individuals at the tooth-jaw contact following the dental line^22,28,29,44,45^. However, *Clevosaurus hadroprodon* has a less developed layer of ‘secondary bone’. Whether this reduced development of ‘secondary bone’ is related to taxonomy or ontogeny is unknown (absence is considered a juvenile feature^46^).

Among sphenodontians, *Clevosaurus hadroprodon* is additionally unusual in its tooth morphology. The teeth are labiolingually compressed, lacking the development of labiolingual features of the marginal dentition common to most other sphenodontians (e.g. a posterolingual crest in *Clevosaurus* species, *Kallimodon*, *Homeosaurus*, *Pleurosaurus*, and *Palaeopleurosaurus*—TRS, pers. obs.; broadly expanded posterior teeth with anteriorly directed concavity teeth in opisthodontians). The absence of labiolingual dental features also occurs in the heterodont teeth of *Whitakersaurus* from the Late Triassic of New Mexico, USA^47^, and *Rebbanasaurus* and *Godavarisaurus*, from the Middle Jurassic–Early Cretaceous of the Kota Formation, India^21^. Among the latter, *Clevosaurus hadroprodon* also shares with *Rebbanasaurus* an extremely deep symphysial margin of the dentary, although differing from the latter in lacking striated tooth crowns.

The presence of a single tooth on the premaxilla of *Clevosaurus hadroprodon* is shared with the sphenodontians *Priosphenodon* and *Sphenodon* as well as the clevosaurs *C*. *brasiliensis*^19,25^, *C*. *bairdi*^18^ and specimens currently attributed to *Clevosaurus* sp. from China^17,22^, differently from the pattern observed in other clevosaurs, such as *C*. *hudsoni*, *C*. *convalis* and *C*. *sectumsemper* that possess 2–4 premaxillary teeth^11,28,48^. In the stem rhynchocephalians *Gephyrosaurus* and *Diphydontosaurus* there are typically five or more small pleurodont teeth on the premaxilla^34,35^, and *Planocephalosaurus* possesses four subpleurodont premaxillary teeth (“semi-pleurodont”^28,34^).

The anterior tusk-like tooth in the premaxilla of *Clevosaurus hadroprodon* is most similar to *C*. *brasiliensis* in being tusk-like (e.g., Hsiou *et al*.^25^; TRS pers. obs.), and not chisel-shaped as in *Sphenodon*, *Priosphenodon*, and *Vadasaurus* (composed of three fused premaxillary teeth^49^). Additionally, an enlarged successional tooth (“canine-tooth”) in the dentary has also been described for *Cynosphenodon* and *Sphenovipera*^23,40^, but these occupy tooth positions posterior to the first tooth. The most similar condition to the enlarged, tusk-like, anteriormost tooth of the dentary in *C*. *hadroprodon* is possibly the enlarged, albeit of a much smaller relative size, anteriormost dentary tooth of *Sphenocondor*. However, in *Sphenocondor* the symphysial portion of the dentary is poorly preserved making it difficult to confirm the exact dimensions of this tooth^27^. A single, large tooth in the first dentary tooth position is also present in *Cynosphenodon huizachalensis*^23,41^. However, in *C*. *huizachalensis* this tooth is conical and preceded by an edentulous gap in adults and in juveniles is preceded by at least three “anterior hatchling teeth” and one “first generation successional tooth”^41^. According to Apesteguía *et al*.^29^ the precise homology between the ‘caniniform’ tooth and other successional teeth remains uncertain in fossil taxa, and in this way, we consider that the tusk-like, anteriormost tooth of the dentary in *C*. *hadroprodon* as a successional tooth that is larger than subsequently successional teeth^29^.

As noted above, although important similarities exist between *Clevosaurus hadroprodon* and Mexican and Indian forms (*Cynosphenodon* and *Rebbanasaurus*), the combination of features in *C*. *hadroprodon* is unique among known sphenodontians and other clevosaurs, thus supporting its designation as a new species of *Clevosaurus*.

## Discussion

The labiolingually compressed marginal dentition of *Clevosaurus hadroprodon* is generally similar to the overall dimensions of the “cut and slice” sphenodontian tooth form^22^ including the marginal teeth of *Clevosaurus* spp. and the sphenodontine sphenodontians^50^. However, *Clevosaurus hadroprodon* differs from these taxa (and nearly all other sphenodontians) in the lack of obvious wear facets on the teeth indicating that the feeding strategy of this new taxon likely did not utilize the intense oral food-processing common to most sphenodontians. The sharply pointed dentition is more similar to that of *Agama*^51^ and juvenile *Uromastyx*^52^—though these acrodontan lizards also show heavy wear facets in the teeth, particularly in older individuals—and suggests a similar arthropod-based diet. It is possible that the specimens of *C*. *hadroprodon* represent juveniles that had not yet been able to achieve significant wear in the marginal dentition, but the presence of “secondary bone” indicates that these specimens had undergone some appreciable ontogenetic development (*sensu* Duffin^46^). The presence of a single, large tooth in the premaxilla and the dentary of *C*. *hadroprodon* is similar to the one observed in *Agama*, but in *C*. *hadroprodon* these teeth are tusk-like (straight and blunt) rather than sharply pointed and recurved. It is possible that such teeth could be used to subdue a prey item, but it is also possible that they served to aid in non-feeding behaviors such as mate competition or defense. The lack of palatal elements precludes assessment of this dentition and associated implications on feeding strategy, mechanism, or diet.

The teeth of *Clevosaurus hadroprodon* represent the oldest occurrence of the typical fully acrodont dentition of sphenodontians anywhere in the world, thus providing insights on the early stages of the development of the acrodont dentition in sphenodontians. In sum, the dental morphology in *C*. *hadroprodon* demonstrates that the presence of a fully acrodont dentition, reduction in premaxillary tooth count and development of large successional teeth on the premaxillae, typical of most sphenodontians, had evolved by the Late Carnian. Conversely, the simple, triangular shape of the marginal teeth in *C*. *hadroprodon* suggests that accessory crests—a feature common to the dentition of most sphenodontians—possibly evolved after the acquisition of the fully acrodont dentition in sphenodontians. But, as stated above, this simple tooth form may be an ontogenetic feature and additional, more complete specimens are needed to verify either of these possibilities.

**Figure 3:**
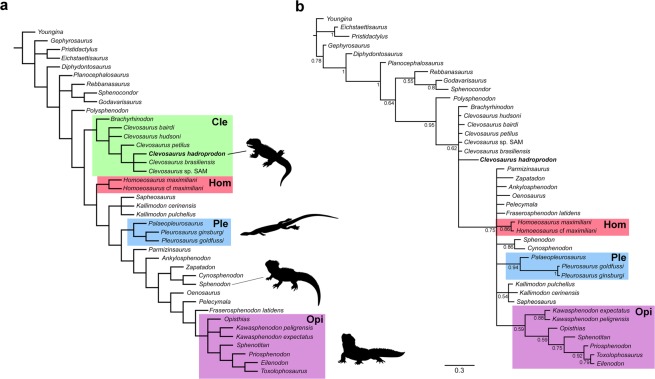
Phylogenetic analyses evidencing the position of *Clevosaurus hadroprodon* sp. nov. within Rhynchocephalia using two distinct optimality criteria. (**a**) Resultant topology of the strict consensus of eight most parsimonious tree (249 steps each) (CI = 0.43; RI = 0.69); (**b**) Majority-rule consensus tree derived from the Bayesian analysis. Nodes values represent posterior probabilities. (Note that Clevosauridae sensu Bonaparte and Sues, was not recovered as a monophyletic group in both analyses and the genus *Clevosaurus* was not recovered as monophyletic in the Bayesian topology). Abbreviations: “Cle”: Clevosauridae; Hom: Homeosauridae; Pleu: Pleurosauridae; Opi: Ophistodontia.

Phylogenetic analysis recovers *Clevosaurus hadroprodon* within Clevosauridae. In a parsimony analysis (Fig. 3a) *C*. *hadroprodon* resolves within the genus *Clevosaurus*, whereas in an analysis utilizing bayesian optimality criterion (Fig. 3b), *C*. *hadroprodon* is recovered in a polytomy comprised of *Brachyrhinodon* and the *Clevosaurus* species. The erection of a new species is justified, although it lacks significant support given: (1) the overall morphological differences between the new species compared to *Clevosaurus* spp. (see comparative osteology section); and (2) the result of the bayesian inference, which does not recover the genus *Clevosaurus* as monophyletic. The poor resolution among the clevosaurids and related taxa in the bayesian topology, in addition to the position of *Polysphenodon* in the parsimony analysis, demonstrates the need for careful and detailed anatomical and systematic revision of Clevosauridae to clarify the interrelationships within these sphenodontians (see also Hsiou *et al*.^25^).

The Carnian age of *Clevosaurus hadroprodon* makes it the oldest known unequivocal sphenodontian from southern Pangaea (Gondwana). Apart from this new record, the former oldest records are the Brazilian *Clevosaurus brasiliensis*^19,24,25,38^ (Norian age; 225.42 ± 0.37 Ma, representing the maximum age of the *Riograndia* AZ, at the top of the Candelária Sequence^33^) and the early opisthodontid sphenodontian *Sphenotitan leyesi*^43^ from the late Norian–Rhaetian of the Quebrada del Barro Formation in Argentina^53,54^ (see Supplementary Information). *Clevosaurus hadroprodon* also represents an early diversification of specialized sphenodontians that occurred within the Gondwanan region of Pangaea prior to the Late Triassic.

**Figure 4:**
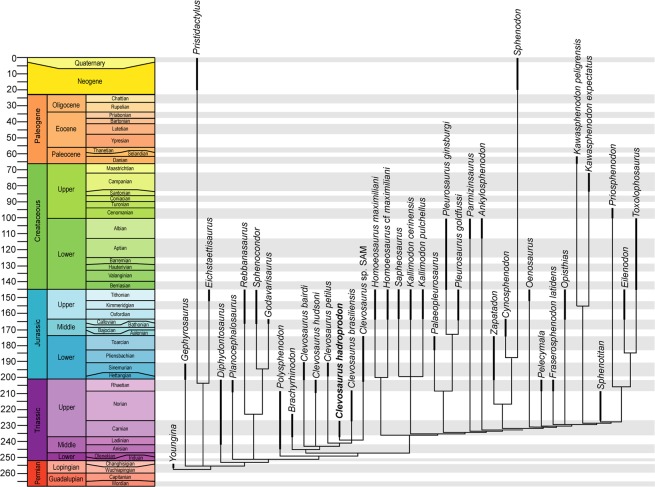
Time–scaled strict consensus topology of Rhynchocephalian relationships (derived from the parsimony analysis). Thickened lines represent the range of age of each taxon according the first appearance date (FAD) and last appearance date (LAD) of the fossil record (see supplementary data 3).

A major component of this early diversification of sphendontians is driven by the origins of clevosaurs, within which *Clevosaurus hadroprodon* figures among the oldest known representatives (Fig. 4), along with *Brachyrhinodon* and *Polysphenodon* from the late Carnian–Norian^55^ of Britain (see also Supplementary Fig. 1 for maximum clade credibility tree and Supplementary Data 2).

The occurrence of Clevosauridae in the late Carnian is notable in that it is approximately 10 my younger than the oldest known rhynchocephalian, cf. *Diphydontosaurus* (“Vellberg jaws” specimens) from the Ladinian (Middle Triassic) of Germany^5^ and only 18 million years younger than the estimated divergence between *Gephyrosaurus* and all other rhynchocephalians^2^. Therefore, clevosaurs comprised the earliest evolving major group of lepidosaurs (with six species that eventually dispersed throughout five different continents), and seem to have developed a widespread geographical distribution very early in their evolutionary history. By the end of the Carnian, clevosaurs were present in at least two distantly located regions of Pangaea that today constitute parts of the United Kingdom and Brazil^18,22,25^. By the Early Jurassic clevosaurs had further dispersed across the fragmenting remains of Pangaea into North America^18^, Asia^17^, and South Africa^16^. As such, clevosaurs represent the earliest group of lepidosauromorphs to achieve such cosmopolitan distribution.

The dentition of *Clevosaurus hadroprodon* represents the oldest occurrence of the typical fully acrodont dentition of sphenodontians anywhere in the world, thus providing insights on the early stages of the development of the acrodont dentition in sphenodontians. For instance, the morphology of *Clevosaurus hadroprodon* suggests that accessory crests that are commonly observed on the dentition of most sphenodontians developed at a later stage in sphenodontian evolution, after the acquisition of the fully acrodont dentition, but not to the exclusion of other dental specializations such as the tusk-like teeth present in this taxon. It further suggests that the reduction in the number of premaxillary teeth and development of large successional teeth on the premaxillae, also typical of most sphenodontians, occurred by the Late Carnian, along with the development of a fully acrodont dentition.

**Figure 5:**
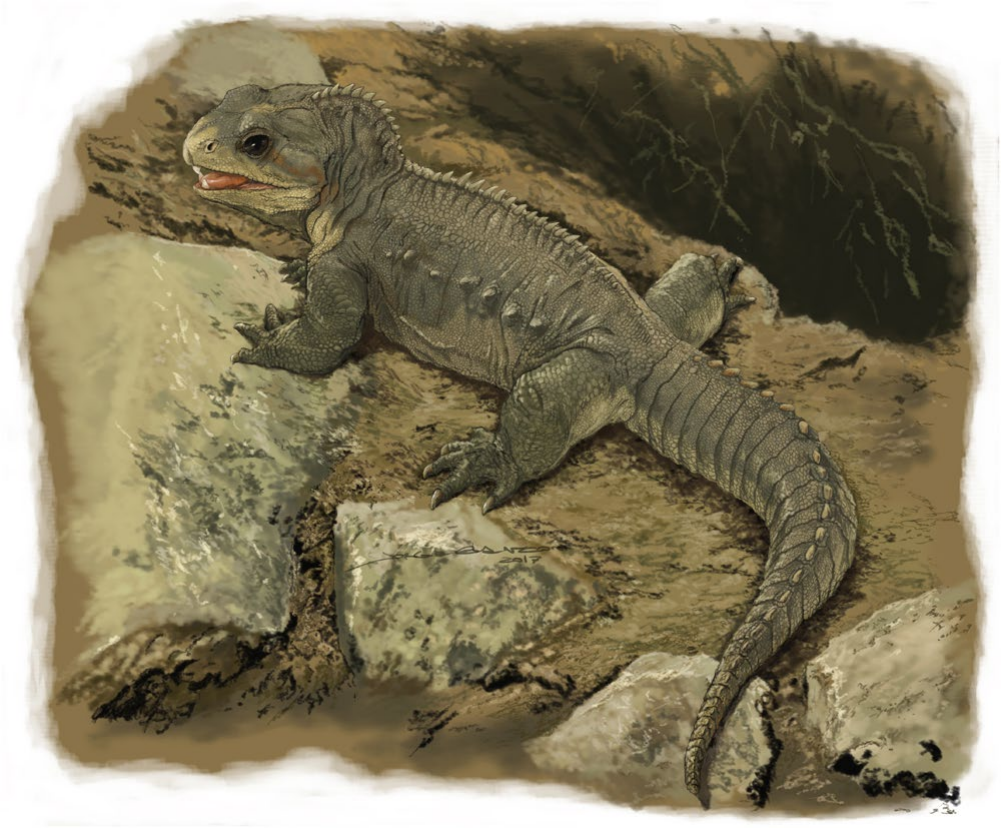
Life reconstruction of *Clevosaurus hadroprodon*. Reconstruction created by Jorge Blanco.

*Clevosaurus hadroprodon* (Fig. 5), along with *Clevosaurus brasiliensis*, indicate that some of the oldest known sphenodontians evolved in South America, and along with Indian taxa, such as *Godavariasaurus* and *Rebbanasaurus*, indicate that some of the earliest diverging members of the Sphenodontia occurred in geographically distant parts of southern Pangaea, during the initial breakup of Gondwana. This illustrates the importance of the role of the Gondwanan lepidosaur fauna in our growing understanding of the earliest stages of sphenodontian evolution and global biogeographic distribution.

## Methods

### Fossil material

Sphenodontian specimens described here are stored at the Museu Municipal Aristides Carlos Rodrigues (MMACR-PV-T, Paleovertebrates-Triassic Collection), Candelária municipality, Rio Grande do Sul State, Southern Brazil. All specimens are incomplete and poorly preserved. The holotype (MMACR PV-027-T) corresponds to an incomplete right premaxilla, an incomplete right maxilla and right lower jaw in occlusion aspect, and left lower jaw located below to these elements. Additional specimens include an incomplete left lower jaw (MMACR PV-028-T) and a fragment of dentary with three isolated teeth (MMACR PV-029-T). Several early and late Mesozoic sphenodontians were used in the comparative morphology, mostly in firsthand examination (by ASH and TRS).

### Type horizon and locality

*Clevosaurus hadroprodon* was collected in the Linha Bernardino outcrop, located in the outskirts of the Candelária municipality (UTM 22J, 324483 E/6717888N, datum WGS 84), Rio Grande do Sul State, Southern Brazil (Fig. 1a–c). The Triassic exposure occurs in a ravine of about six meters depth composed of fine to medium-grained sandstones, intercalated with mudstone layers and conglomerates (Fig. 1b). Carbonate concretions occur along most of the section, and the fossils tend to occur in association with the concretions. The specimens referred to *Clevosaurus hadroprodon* were originally embedded in concretions, abundant at the lowermost conglomeratic level of the outcrop. Other specimens, representing other taxa, were collected in the fine sandstone levels, also associated with concretions. They include partial lower jaws and some postcranial elements (e.g., humerus) referred to the traversodontid cynodont *Exaeretodon riograndensis*, partial skull referred to the probainognathian cynodont *Trucidocynodon* sp., and fragments of long bones of a dinosaur specimen, among other indeterminate materials. A fragment of a femur resembles the early dinosaur *Pampadromaeus barberenai*^56,57^; although further preparation and comparisons are needed to clarify its taxonomy. The uppermost portion of the exposure consists of pinkish non-fossiliferous massive sandstone (Fig. 1b). The presence of specimens referred to *Exaeretodon*, *Trucidocynodon* and a *Pampadromaeus*-like form allows correlation of this outcrop as part of the *Hyperodapedon* AZ, at the base of the Candelária Sequence. The fossil samples from this outcrop are relatively scarce in comparison to other Triassic localities in the state of Rio Grande do Sul and a precise estimation of its relative age is only tentative. *Exaeretodon*, *Trucidocynodon* and a *Pampadromaeus*-like form have also been recovered from the Janner Site, in the municipality of Agudo. At this locality these taxa are part of a more diverse fauna including probainognathian (e.g. *Trucidocynodon riograndensis*) and traversodontid (*Exaeretodon riograndensis*) cynodonts^58,59^, dinosaurs and rhynchosaurs. Based on its faunal content and its relationships with other outcrops from southern Brazil, the Linha Bernardino site can be also correlated to the Ischigualasto Formation (Ischigualasto-Villa Unión Basin, Argentina^60,61^). A recent analysis provided a high-precision U-Pb zircon geochronology to some sections of south Brazil Triassic exposures, including a typical *Hyperodapedon* AZ locality (i.e. Cerro da Alemoa)^33^. The recovered weighted mean ^206^Pb/^238^U date of 233.23 ± 0.73 Ma is slightly older than the datum for the Ischigualasto Formation^33^. Nonetheless, the combined data suggest that the age of the faunal association of the Linha Bernardino site is more likely Late Carnian (MCL, pers. comm.).

### Phylogenetic analyses and time scaled–tree

#### Taxon scoring changes

*Sophineta* was deleted from the dataset because only some of the skull and mandible elements can be confidentially linked to a single taxon, with all the referred postcranial material lacking any anatomical connection to the skull and mandible elements (TRS pers. obs.). Since deletion of all postcranial scorings would highly reduce the usefulness of this taxon as an outgroup by re-scoring several cells with missing data, we deleted this taxon altogether from the analysis.

All specimens attributed to *Clevosaurus brasiliensis* were merged into a single operational taxonomic unit (OTU), since all of them have been subject of a recent revision of this species and confidently attributed to a single taxon^25^. We agree with the interpretation of lack of diagnostic features to separate *C*. *wangi*, *C*. *mcgilli* and *C*. *petilus*^18,22^, and thus we follow Sues *et al*.^17^ in considering all three Chinese clevosaurs as a single OTU, represented by *C*. *petilus*.

#### Character changes

Characters 62 and 63 were scored with missing data (“?”) for all taxa and were therefore removed from the present dataset. New character 68 added.

#### Character-state changes

New states added herein: Ch. 47, state 3.

### Taxon scoring and re-scoring

Ch. 8. *Clevosaurus* sp. SAM:?→0; *Clevosaurus brasiliensis*: 0/1→0 (only specimen with a “deep” maxilla has the posterior end of the maxilla incomplete, with the process extending ventral to the broken jugal).

Ch. 12. *Clevosaurus petilus*: 0→1; *Brachyrhinodon*: 0→?.

Ch.19: *Clevosaurus brasiliensis*: 1→0.

Ch.21: *Clevosaurus* SAM: 2→? (jugal posterior border seemingly broken); *Clevosaurus petilus* 1→? (jugal posterior border poorly preserved).

Ch.33. *Clevosaurus latidens*: 3→? (symphysis not preserved); *Sphenotitan*: 3→0; *Oenosaurus*: 2/3→2; *Planocephalosaurus*: 1→2; *Pelecymala*: 1→2. *Beachyrhinodon* 1→? (symphysis poorly preserved); *Clevosaurus hudsoni*: 1→2; *Clevosaurus convalis*: 1→0; *Clevosaurus bairdi*: 1→2; *Clevosaurus* SAM:?→2; *Clevosaurus brasiliensis*: 1→2.

Ch. 36: *Clevosaurus* SAM:?→0; *Oenosaurus*: 1→?.

Ch. 44: Pleurosaurus goldfussi 2→ 3 (BSPG 1925 I 18 [holotype], BSPG 1978 I 7); Pleurosaurus gingsburi 2/3→3 (MNHN 1983-4-CNJ 67 [holotype], BSPG 1977 XIX 40); Palaeopleurosaurus posidoniae 2→3 (SMN 50721).

Ch.47: *Opisthias*: 1→2.

Ch. 50. *Brachyrhinodon* 1→?.

Ch. 51. *Brachyrhinodon* 1→?.

Ch. 57: *Clevosaurus brasiliensis*: 0→?.

Ch. 69: Sphenotitan: 2→?.

Ch. 72: *Clevosaurus bairdi*: 2→3; *Clevosaurus hudsoni*: 2→1; *Clevosaurus brasiliensis*: 2→1; *Beachyrhinodon* 2→?; *Clevosaurus convalis*: 2→?.

### Rogue taxon identification

An initial set of analyses (Analysis 1) was conducted with all taxa initially available in the data set. This analysis resulted in consensus trees with important sectors of the tree highly unresolved (results available in Supplementary Information). Therefore, we conducted a rogue taxon identification analysis using the RogueNaRok algorithm, which can improve tree accuracy and resolution when specific wildcards are the main factor contributing to poorly resolved nodes^62^. We also utilized the TNT pruning trees algorithm to detect wild card taxa in specific nodes of the consensus tree inferred with maximum parsimony. The results indicate that the removal of *Clevosaurus convalis*, *Sphenovipera* and *Theretairus* would highly improve phylogenetic resolution. A second set of analyses were conducted after removal of those three taxa, which yielded better trees that are reported in the main text.

### Phylogenetic analyses

Maximum parsimony analysis was conducted in TNT v. 1.1^63^ using 100 tree replicates obtained by random addition sequence (RAS), and searching for new tree topologies with tree bisection and reconnection (TBR), saving 100 trees per replication. For Analysis 1, a total of 907 most parsimonious trees (MPTs) were obtained with 258 steps each. After removal of the three rogue taxa, the 40 remaining taxa were analyzed in a new set of analyses (Analysis 2). In Analysis 2, we recovered a total of eight MPTs with 249 steps each.

The Bayesian inference analysis was conducted using Mr. Bayes v. 3.2.6^64^ using the Mkv model for morphological data^65^, and with rate variation across characters sampled from a gamma distribution. Each analysis was performed with two independent runs of 1 × 10^7^ generations each, with eight chains per run and four swaps attempted per swapping generation. The relative burn-in fraction was set to 50% and the chains were sampled every 50 generations. The temperature parameter for the four chains in each independent run was set to 0.02. Convergence of independent runs was assessed through the average standard deviation of split frequencies (ASDSF < 0.01) and potential scale reduction factors (PSRF ≈ 1 for all parameters) calculated at the end of the Bayesian runs. We used Tracer v. 1.6^66^ to determine whether the runs reached stationary phase and to ensure that the effective sample size (ESS) for each parameter was greater than 200.

The strict consensus tree calculated from the eight MPTs from the parsimony Analysis 2 was time–scaled using the package *strap*^67^ for the software R^68^. The range of time for each taxon was delimited consulting the relevant literature (see Supplementary Data 3 and 4). It was employed in the scaling, the *Brussate* parameter^69^, in which all branch lengths are shared equally among the tree, in order to avoid zero values (*i*.*e*. only retaining positive length for each branch).

## Supplementary information

Supplementary material

## Data Availability

The phylogenetic analyses, time scaled–tree and geological unit and time range references of the Rhynchocephalia taxa data of this study are available as Supplementary Information.
