# Author Correction: A New Clevosaurid from the Triassic (Carnian) of Brazil and the Rise of Sphenodontians in Gondwana

**DOI:** 10.1038/s41598-021-88869-2

**Published:** 2021-05-06

**Authors:** Annie S. Hsiou, Randall L. Nydam, Tiago R. Simões, Flávio A. Pretto, Silvio Onary, Agustín G. Martinelli, Alexandre Liparini, Paulo R. Romo de Vivar Martínez, Marina B. Soares, Cesar L. Schultz, Michael W. Caldwell

**Affiliations:** 1 Laboratório de Paleontologia, Universidade de São Paulo, Ribeirão Preto, São Paulo Brazil; 2 Arizona College of Osteopathic Medicine and Department of Anatomy, College of Graduate Studies, Midwestern University, Glendale, AZ 85308 USA; 3 Department of Biological Sciences, University of Alberta, Edmonton, Canada; 4 Present Address: Department of Organismic and Evolutionary Biology, Museum of Comparative Zoology, Harvard University, Cambridge, MA 02138 USA; 5 CAPPA - Centro de Apoio à Pesquisa Paleontológica da Quarta Colônia, Universidade Federal de Santa Maria, São João Do Polêsine, Brazil; 6 CONICET-Sección Paleontología de Vertebrados, Museo Argentino de Ciencias Naturales ‘Bernardino Rivadavia’, Buenos Aires, Argentina; 7 Laboratório de Paleontologia de Vertebrados, Universidade Federal do Rio Grande do Sul, Porto Alegre, Brazil; 8 PIBi-Lab – Laboratório de Pesquisas Integrativas em Biodiversidade, Departamento de Biologia, Universidade Federal de Sergipe, São Cristóvão, Sergipe Brazil; 9 Department of Earth and Atmospheric Sciences, University of Alberta, Edmonton, Canada

Correction to: *Scientific Reports* 10.1038/s41598-019-48297-9, published online 14 August 2019

The original version of this Article did not include evidence of registration in ZooBank within the work itself, as required by Article 8.5.3 of the International Code of Zoological Nomenclature^1^. Therefore, the details of the newly proposed species name *Clevosaurus hadroprodon* and a description of the Nomenclatural Acts were not available at the time of publication.

This published work and the Nomenclatural Acts it contains have been registered in ZooBank with the LSID urn:lsid:zoobank.org:pub:02B4B0AF-B553-410B-94B1-76D7AE277FAE for this publication and urn:lsid:zoobank.org:act:971AD911-D5FB-460B-91AF-25B3D973873E for the species.

The original Article has been corrected.
