# Supplementary material for: A New Clevosaurid from the Triassic (Carnian) of Brazil and the Rise of Sphenodontians in Gondwana
Source: Sci Rep. 2019 Aug 14;9:11821. doi: 10.1038/s41598-019-48297-9 (PMC6694142; doi:10.1038/s41598-019-48297-9)
Supplement: Supplementary file 1 — Supplementary material [file 41598_2019_48297_MOESM1_ESM.pdf]

Manuscript:

A NEW CLEVOSAURID FROM THE TRIASSIC (CARNIAN) OF BRAZIL AND THE RISE OF SPHENODONTIANS IN GONDWANA

Annie S. Hsiou[1*], Randall L. Nydam[2], Tiago R. Simões[3,4], Flávio A. Pretto[5], Silvio Onary[1] Agustín G. Martinelli[6,7], Alexandre Liparini[8], Paulo R. Romo de Vivar Martínez[7], Marina B. Soares[7], Cesar L. Schultz[7], & Michael W. Caldwell[3,9]

[1]Laboratório de Paleontologia, Universidade de São Paulo, Ribeirão Preto, São Paulo, Brazil. [2]Arizona College of Osteopathic Medicine and Department of Anatomy, College of Graduate Studies, Midwestern University, USA. [3]Department of Biological Sciences, University of Alberta, Edmonton, Canada. [4]Current address: Department of Organismic and Evolutionary Biology, Museum of Comparative Zoology, Harvard University, Cambridge, MA 02138, USA. [5]CAPPA - Centro de Apoio à Pesquisa Paleontológica da Quarta Colônia, Universidade Federal de Santa Maria, São João do Polêsine, Brazil. [6]CONICET-Sección Paleontología de Vertebrados, Museo Argentino de Ciencias Naturales 'Bernardino Rivadavia', Buenos Aires, Argentina. [7]Laboratório de Paleontologia de Vertebrados, Universidade Federal do Rio Grande do Sul, Porto Alegre, Brazil. [8]PIBi-Lab – Laboratório de Pesquisas Integrativas em Biodiversidade, Departamento de Biologia, Universidade Federal de Sergipe, São Cristóvão, Sergipe, Brazil. [9]Department of Earth and Atmospheric Sciences, University of Alberta, Edmonton, Canada. Correspondence and request for materials should be addressed to A.S.H. (email: anniehsiou@ffclrp.usp.br

Supplementary Image 1: Maximum clade credibility tree derived from the post burn-in sample of posterior trees. Node values represent posterior probabilities.

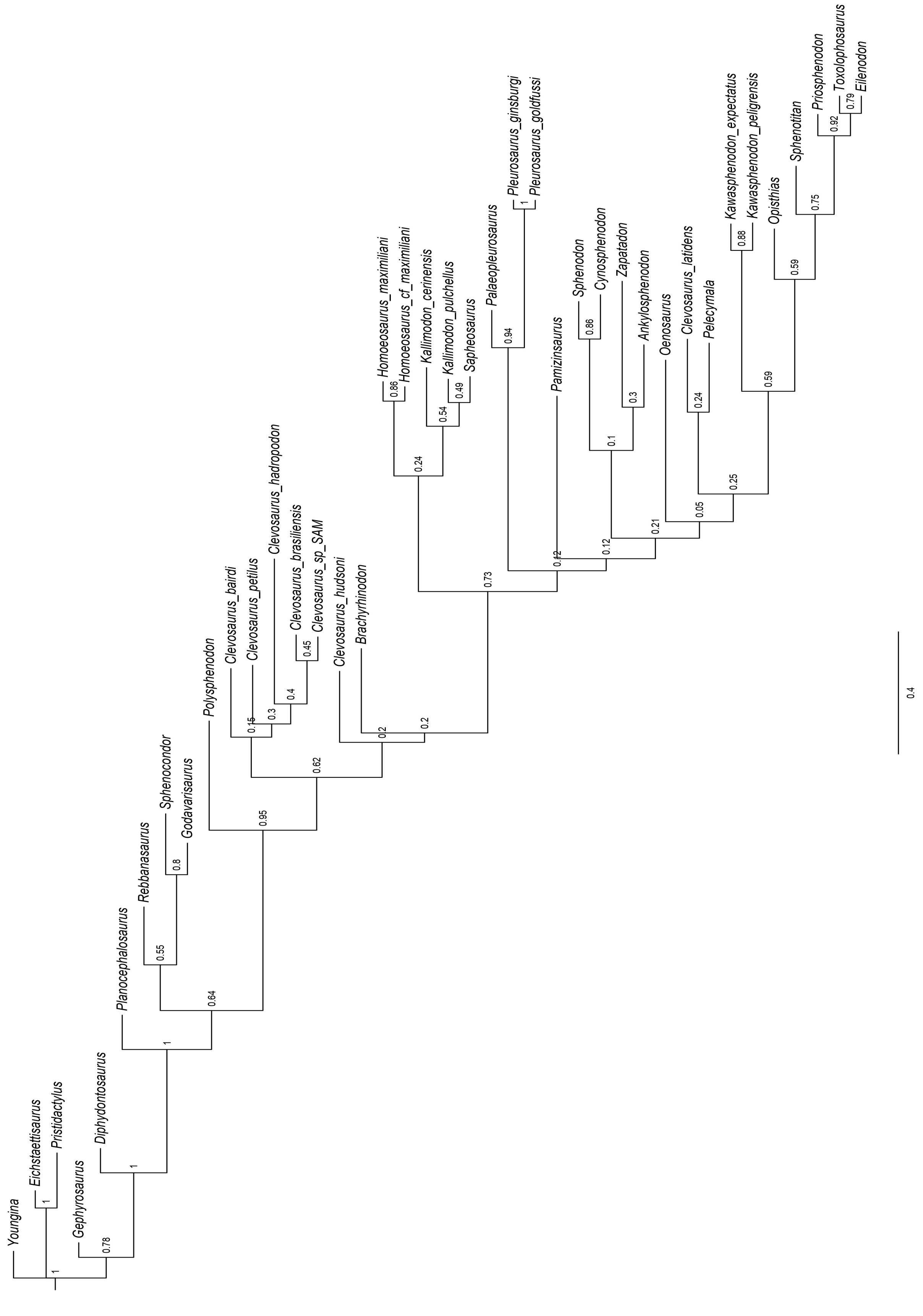

Supplementary Data 1

Table 1: Morphological table of comparison regarding dental and dentary features between some selected lepidosaurs lineages.

Feature	Sphenodontidae	Chamaeleontidae	Uromastycinae	Agamidae*
1. Posterior dentary and maxillary tooth series entirely placed apically on the jaw bone	Yes	Yes	No	Rarely
2. Anterior dentary tooth series entirely placed apically on the jaw bone	Yes?	Yes	No	No
3. Anteriormost canine-like tooth on dentary	Variable	No	No	Variable
4. Secondary bone deposition on jaws	Variable	No	No	No
5. Deep dentary symphysis	Yes	Yes	Yes	Variable
6. Vertically oriented dentary symphysis	Variable	No	Yes	Variable
7. Deep ventral crest of the dentary medial wall through whole extension of dentary	Yes	Yes	Yes	Very common
8. Shallow Meckelian canal	Yes	No	No	No

* Non-uromasticine agamids, based on *Agama, Draco, Pogona, Physignathus* and *Leiolepis*.

Supplementary Data 2

Assembled dataset in Nexus extension used for the phylogenetic analyses (including the three rogue taxa). Details about the scoring and analyses in the Material and Methods section.

```
xread
73 43
Youngina
        000000000000000000020000000?0000?0?000000020000000000000?10?00010?0?0000
Eichstaettisaurus
        1011000010?011000000??000???00?0????????????????????????????????0?000?
Pristidactylus
        1111100001100110001?0001000?000?00?000000000200000????200?11100010?0?0001
Gephyrosaurus
        00000000000001100001200000000010100?000001000200000000000?10200010?0?0001
Diphydontosaurus
        1000000010000100000120100000010100001000111120010000000000121?000010?0000
Planocephalosaurus      000000001000011001012020000101012100001012 12?0[0
1]2010000110121111[1 2]00?10?0010
Rebbanasaurus ???????01??0??????????????????0?3102?????212200201001??101???[0
1]110001100??
Godavarisaurus ??????1?????????????????????????0110[0      2]?????212200[1      2]01021??100???[0
1]100101100??
Homoeosaurus_maximiliani      1[0     1]0000011000?0000[1     2]11??2001???10111101?11121201[0
1]21202??21112221?????0?000?
Homoeosaurus_cf_maximiliani 11[0 1][0 1]??????0??00?0?11?02??1?????[0 1]1???1?11121[2 3]01[0
1]??20[1 2]??2?????21???????????
Palaeopleurosaurus      0111000010001011120020211011010011 1?1?1[0
1]1213011211020021012100 22???0?0001
Pleurosaurus_goldfussi 0110?0001100?0011210002[1
2]1010010201 10??0012130012000210211 0?20022???0?0000
Pleurosaurus_ginsburgi
        0110?0001?00?001????0?????????0?0110?????2130012000??0?110120022???0?0000
Kallimodon_pulchellus 01110?011?00?0011211112001???10[0
1]11021?101212011212 02??2001221122???0?00??
Kallimodon_cerinensis ???????????????????1??2010?????[0           1]110?1?[0           1]?1212??[0
1]2120200200022 1122?????00??
Sapheosaurus    111100?1?0???001121121 1001????0[0                                        1]11021?10[0
1]???01?21?0???2???221122?????????
Pamizinsaurus   ??????1???0?????????????211??0?0?[1                                         2]11?1?11?2120?[0
1]2?102???101???12???10?????
Zapatadon       10?01??01??1??1?1?1?102210101[0                                         1]1?1112??1??2120?[0
1]??102??2??????12????0????0
Sphenodon       11111001110010011111212221110111211121111121211[0       1]21102112100012[1
2]22100100011
Cynosphenodon
        ??????????????????????????????1?2112??1??2121112110????100???112100110011
Opisthias       ????????????????????????????????3111??[0
1]??212012??20????112???122???0?10??
Toxolophosaurus
        ??????????????????????????????1?31111?0??213012????????112???222???0?01??
Eilenodon       ??????????1???????????????????12311112022213012 2?21210?112???222?????0122
Priosphenodon  0110011111111001111101220100011231111202221301221212103112 2[1
2]2222???0?0032
```

Ankylosphenodon
???1?????1????????????????????1?111?1?111?1?01????0????0?0221121???0?0???
Sphenocondor ????????????????????????????????1?1?1?1?1212200????????0?0???0[1
2]?0101100??
Sphenovipera ??????????????????????????????1?21021???1212211????????112???1[1
2]211?1?10??
Theretairus ??????????????????????????????1?210??????21221?????????1?????12?10?0?00??
Kawasphenodon_expectatus
??????????????????????????????1??????????212?10?????1??112???[1 2]???????02??
Kawasphenodon_peligrensis ??????????????????????????????1??????????21[2
3]?10????????112????????????02??
Oenosaurus 20???0?1??0?10111????02201????112?1?111112??0?[0
1]????2?02000???2?2???0?00?1
Sphenotitan
21110111??11?11111121220?10011201110200121301221211100 11????1221000???0?
Pelecymala ?????001???????????????????????1?2????????212?12??10????10?????221??????0?
Polysphenodon 2000???11100?0000001?00??1????01???????01211?10211?1??1?????1??????????1?
Brachyrhinodon
211111011100?000000120120?????00?1101?10121201121??1001?????11??????????1
Clevosaurus_latidens
??????1???????????????????????1??110?????212??2??1??????0?????221????????
Clevosaurus_convalis
?????1????????????????0??????????0110??1??2120111?20??0?100???1?2??00?00??
Clevosaurus_hudsoni
21111111110100010001202101000000211011101212011212010011012111112??00?0011
Clevosaurus_bairdi
21111111101?001000120??010?00012??00110?21?010212?1??11?02??1?2??00?0031
Clevosaurus_petilus
1111111110100?00001?02101000001?1?011101212?1021201 1001?0?????????0??0??
Clevosaurus_sp_SAM
?????110?101?0?????1??2??????0??12??01???1?1????21??1????????????????0?????
Clevosaurus_brasiliensis
111111011010000010120210 1??00012100111012120112100 10000?0???1?2??00?0?11
*Clevosaurus*_hadroprodon
?????1????????????????????????????1?0?????212?032100????000?????21001?00??
;

proc /;
comments 0
;

Supplementary Data 3

Table showing the geological unit and time range references of the here studied Rhynchocephalia taxa. The time range of each taxon was applied in our time-scaled tree analysis.

Taxon	Geology Unit	Age	Country/Supercontinent	References
Clevosaurus hadroprodon	Linha Bernardino locality, Candelária municipally, Santa Maria Formation (Supersequence Santa Maria, Candelária Sequence, *Hyperodapedon* Zone), Rosário do Sul Group, Paraná Basin	Late Triassic (late Carnian)	Brazil – Gondwana	Zerfass et al., (2003)<br>Horn et al., (2014)
Clevosaurus brasiliensis	Linha São Luis locality, Faxinal do Soturno municipally, Caturrita Fomation (Supersequence Santa Maria, Candelária Sequence, *Riograndia* Zone), Rosário do Sul Group, Paraná Basin	Late Triassic (early Norian - 225.42±0.37 Ma maximum age)	Brazil - Gondwana	Zerfass et al., (2003)<br>Horn et al., (2014)<br>Langer et al., (2018)
Clevosaurus hudsoni	Black Rock Limestone Subgroup and/or Gully Oolite Formation (Cromhall Quarry)- Carboniferous Limestone of Early Carboniferous (Mississippian)	Late Triassic (late Rhaetian)	UK – Laurasia	Morton et al., (2017)
Clevosaurus convallis	Pant 4 fissure in Pant Quarry, Glamorgan, South Wales, one of a series of Lower Jurassic fissure Fills (Carboniferous Limestone)	Early Jurassic	UK - Laurasia	Säilä (2005)
Clevosaurus bairdi	McCoy Brook Formation, Fundy Rift Basin	Early Jurassic (Hettangian-Pliensbachian)	Canada - Laurasia	Tanner (1996)
Clevosaurus petilus	Dull Purplish Beds, Lower Lufeng Formation, Lufeng Basin	Early Jurassic (Sinemurian)	China – Laurasia	Wu (1994)<br>Luo and Wu (1994)
Brachyrhinodon	Lossiemouth Formation	Late Triassic (222.8 Mya, late Carnian)	Scotland – Laurasia	Fraser & Benton, (1989)<br>Gradstein et al., (2004, 2012)<br>Jones et al., (2013)
Polysphenodon	Keuper Formation, Keuper Sandstone Group	Late Triassic (Canian-Norian)	Germany – Laurasia	Fraser & Benton, (1989)
Gephyrosaurus bridens	Pontalun quarry, near Bridgend in South Glamorgan, St. Bride's island	Early Jurassic (Late Hettangian-Early Sinemurian age)	UK - Laurasia	Evans, (1980)
Gephyrosaurus evansae	Rhaetian fissure fills in Carboniferous	Late Triassic (Rhaetian)	UK - Laurasia	Whiteside et al., (2017)

	Limestone, Holwell quarry, Somerset			
cf. *Diphydontosaurus*	Lower Keuper, Erfurt Fomation, Layer 6 of Schoch Vellberg locality	Middle Triassic (239-240 Mya, Ladinian)	Germany – Laurasia	Gradstein et al., (2012)<br>Jones et al., (2013)
Diphydontosaurus avonis	Tytherington Quarry, Lower Carboniferous, Black Rock Limestone, South Gloucestershire	Late Triassic (Rhaetian)	UK - Laurasia	Whiteside, (1986)
Rebbanasaurus jaini	Upper Member of the Kota Formation, Upper Gondwana Group	Middle Jurassic (Callovian)/Early Cretaceous (Barriasian)	India – Gondwana	Vijaya & Prasad, (2001)<br>Prasad & Manhas, (2007)
Godavariasaurus lateefi	Upper Member of the Kota Formation, Upper Gondwana Group	Middle Jurassic (Callovian)/Early Cretaceous (Barriasian)	India – Gondwana	Vijaya & Prasad, (2001)<br>Prasad & Manhas, (2007)
Planocephalosaurus robinsonae	Black Rock Limestone Subgroup and/or Gully Oolite Formation (Cromhall Quarry) - Carboniferous Limestone of Early Carboniferous (Mississippian)	Late Triassic (late Rhaetian)	UK – Laurasia	Morton et al., (2017)
Paleopleurosaurus posidoniae	P. Kirchmann Quarry in Saatswald Ohmden near Holzmaden, Posidonia Shale Formation,	Early Jurassic (Toarcian)	Germany – Laurasia	Carroll, (1985)
Sphenodon punctatus	Bannockburn Formation, Manuherikia Group, St Bathans Fauna	Early Miocene (19–16 Mya)	New Zealand	Jones et al. (2009)<br>Worthy et al. (2006, 2007)
Ankylosphenodon pachyostosus	Tlayua Quarry, Locality No. 2432 Cantera Tlayua-IGM, level 'ZlX. Middle member of the Tlayua Formation	Early Cretaceous (Middle to Late Albian)	Mexico – Laurasia	Reynoso (1997, 2000)
Oenosaurus muehlheimensis	Mörnsheim Formation, ‘‘Krautworst Naturstein’’ quarry, Mühlheim	Early Triassic (Lower Tithonian)	Germany – Laurasia	Rauhut et al. (2012)
Pamizinsaurus tlayuaensis	Tlayua Quarry, Locality No. 2432 Cantera Tlayua-IGM, level 'ZlX. Middle member of the Tlayua Formation	Early Cretaceous (Middle to Late Albian)	Mexico – Laurasia	Reynoso (1997)
Zapatadon ejidoemis	Lower part of the La Boca Formation, Tierra Buena, western of Huizachal Canyon	Late Early Jurassic	Mexico – Laurasia	Reynoso & Clarck (1998)<br>Fastovsky et al., (2005)
Priosphenodon avelasi (Kaikaifilusaurus)	Upper layers of the Candeleros Formation, ‘La Buitrera’ fossil quarry, Cerro Policía	Late Cretaceous (Cenomanian – Turonian)	Argentina – Gondwana	Apesteguía & Novas (2003)
Priosphenodon minimus	Upper part of the La Paloma Member	Early Cretaceous (Lower Albian)	Argentina – Gondwana	Apesteguía & Carbadilho, (2014)

	Cerro Barcino Formation, Chubut Group, Central Patagonia.			
Sphenotitan leyisi	Upper layers of the Quebrada del Barro Formation, El Carrizal Basin, 'Balde de Leyes' fossil locality	Late Triassic – Late Norian–Rhaetian	Argentina – Gondwana	Martínez et al. (2013)<br>Martínez & Apaldetti, (2017)
Toxolophosaurus cloudi	Kootenai Formation, Silver Bow County, Montana	Early Cretaceous	United States – Laurasia	Throckmorton et al. (1981)
Eilenodon robustus	Salt Wash Member of the Morrison Formation, Colorado	Late Jurassic	United States – Laurasia	Rasmussen & Calliston (1981)
Opisthias rarus	Morrison Formation "Quarry 9," Como Bluff, Albany County, Wyoming	Late Jurassic	United States – Laurasia	Gilmore (1909)<br>Carrano & Velez-Juarbe (2006)
Kawasphenodon peligrensis	Upper part of the Hansen Member of the Salamanca Formation, Punta Peligro locality	Early Palaeocene, Peligran South American Land Mammal Age, (SALMA)	Argentina – Gondwana	Apesteguía et al. (2014)
Kawasphenodon expectatus	Mid layers of the Los Alamitos Formation, Estancia 'Los Alamitos' locality	Late Cretaceous (Late Campanian–Early Maastrichtian)	Argentina – Gondwana	Apesteguía (2005)
Pelecymala robustus	Karstic fissures in Dinantian limestones. Cromhall Quarry, south Gloucestershire.	Late Triassic (Rhaetian)	UK – Laurasia	Fraser (1986)<br>Whiteside & Marshall, 2007
Sigmala sigmala	Karstic fissures in Dinantian limestones. Cromhall Quarry. south Gloucestershire.	Late Triassic (Rhaetian)	UK – Laurasia	Fraser (1986)<br>Whiteside & Marshall, 2007
Homoeosaurus maximiliani	Solnhofen Limestone, Kelheim, Solnhofen & Eiehtatt, Bavaria	Late Jurassic (Lower Tithonian-Kimmeridgian)	Germany - Laurasia	Meyer, 1845<br>Cocude-Michel (1963)
Homoeosaurus cf. maximiliani	Calcaire lithographique, Cerin (Ain)	Late Jurassic (*Kimmeridgian*)	France - Laurasia	Cocude-Michel (1963)
Sphenocondor gracilis	Lower Member of the Cañadón Asfalto Formation, Queso Rallado locality	Middle Jurassic (Callovian)	Argentina – Gondwana	Rougier et al., 2007;<br>Apesteguía, Gómez & Rougier (2012)
Cynosphenodon huizachalensis	Lower part of La Boca Formation, Huizachal Canyon	Middle Jurassic	México – Laurásia	Reynoso (1996)
Kallimodon cerinensis	Calcaire Lithographique, Cerin (Ain)	Late Jurassic (Kimmeridgian)	France –Laurasia	Cocude-Michel (1963)<br>Reynoso, (1996)
Clevosaurus sp. SAM	Elliot Formation or from the Clarens Formation (upper part of Stormberg Group	Early Jurassic	South Africa-Gondwana	Sues & Reiz, (1995)

Supplementary Data 4

Script employed to generate the time-scale tree in software R.

```
#This is the script used in order to generate a calibrate tree

#Installing the strap package from Bell and Lloyd (2014)
install.packages(c("geoscale", "strap"), dependencies=TRUE)

#Loading the package
library(strap)

#Set the dir file (in your PC)

#Loading the age file
super.age <- read.table("ages.txt", header=T)

#Testing the ages file
super.age

#Loading the Phylogenetic Tree (in Nexus file)
super.tree <- read.nexus("Rhyncho.nex")

#Checking the file
super.tree

#using the time-scaling function DatePhylo with default options
ts.tree.stan <- DatePhylo(super.tree, super.age)

#Applying the time-scaling function DatePhylo with Brusatte method
ts.tree.brus <- DatePhylo(super.tree, super.age, rlen=1, method="equal")

#Plotting and comparing the different topologies
plot(super.tree)
plot(ts.tree.stan)
plot(ts.tree.brus)

#Rooting the stratigraphic age
#we can access typing:
ts.tree.stan$root.time
ts.tree.brus$root.time

#Plotting our tree against the current geologic time scale (Gradstein et al. 2012)
geoscalePhylo(ts.tree.stan)
geoscalePhylo(ts.tree.brus)

#Saving and exporting the file as a .tre or .nex
write.tree(ts.tree.stan, file="timetre-standard.tre")
```

```
write.nexus(ts.tree.stan, file="timetree-standard.nex")
write.tree(ts.tree.brus, file="timetree-brus.nex")
write.nexus(ts.tree.brus, file="timetree-brus.nex")

#Installing package Paleotree
install.packages("paleotree", dependencies = TRUE)
library(paleotree)
super.ts.tree <- timePaleoPhy(ts.tree.brus, super.age, "mbl", 2)
pdf("super_tree_3.pdf", width=10, height=7)
  geoscalePhylo(super.ts.tree)
dev.off()

#Export PDF
pdf("super_tree_1.pdf", width=10, height=7)
  geoscalePhylo(super.ts.tree)
dev.off()

#plot with terminal taxa stratigraphic distribution, generating a final analysis tree against the stratigraphic (with terminal ranges)
pdf("super_tree_4.pdf", width=10, height=7)
  geoscalePhylo(ladderize(ts.tree.brus, right=FALSE), super.age, cex.ts=0.5)
dev.off()
```

Supplementary references

Apesteguía S, Carballido JL. A new eilenodontine (Lepidosauria, Sphenodontidae) from the Lower Cretaceous of central Patagonia. *Journal of Vertebrate Palaeontology* **34**: 303–317 (2014).

Apesteguía S, Gómez RO, Rougier GW. 2014 The youngest South American rhynchocephalian, a survivor of the K/Pg extinction. *Proc. R. Soc. B* **281**: 20140811.

Apesteguía S, Novas FE. Large Cretaceous sphenodontian from Patagonia provides insight into lepidosaur evolution in Gondwana. *Nature* **425**: 609–612. (2003).

Apesteguía, S. A Late Campanian sphenodontid (Reptilia, Diapsida) from northern Patagonia. *C.R. Palevol* **4**, 663-669 (2005).

Carrano, M.T., Velez-Juarbe, J. Paleoecology of the Quarry 9 vertebrate assemblage from Como Bluff, Wyoming (Morrison Formation, Late Jurassic). *Palaeogeography, Palaeoclimatology, Palaeoecology* **237**, 147-159 (2006).

Carroll, R. A Pleurosaur from the Lower Jurassic and the taxonomic position of the Sphenodontida. *Paleontographica Abt. A* **189**, 1-28 (1985).

Cocude-Michel, M. *Les Rhynchocéphales et les sauriens des calcaries lithographuques (Jurassique supérieur) D'Europe Occidentale*. Ph.D. thesis, Faculté des Sciences de L'Université de Nancy. pp.223 (1963).

Evans, S. *The skull of a new eosuchian reptile from the Lower Jurassic of South Wales*. 203–264 (1980).

Fraser, N. C. & Benton, M. J. The Triassic reptiles *Brachyrhinodon* and *Polysphenodon* and the relationships of the sphenodontids. *Zool. J. Linn. Soc.* 96, 413–445 (1989).

Horn, B. L. D. et al. A new third-order sequence stratigraphic framework applied to the Triassic of the Paraná Basin, Rio Grande do Sul, Brazil, based on structural, stratigraphic and paleontological data. *J. South Am. Earth Sci.* **55**, 123–132 (2014).

Jones, M. et al. Integration of molecules and new fossils supports a Triassic origin for Lepidosauria (lizards, snakes, and tuatara). *BMC Evol. Biol. 13*, 208 (2013).

Jones, M.E.H., Tennyson, A.J.D., Worthy, J.P., Evans, S.E., Worthy T.H.A sphenodontine (Rhynchocephalia) from the Miocene of New Zealand and palaeobiogeography of the tuatara (Sphenodon). *Proceedings of the Royal Society B. Proc. R. Soc. B.* doi:10.1098/rspb.2008.1785 (2009).

Klein, C. G., Whiteside, D. I., de Lucas, V. S., Viegas, P. A. & Benton, M. J. A distinctive Late Triassic microvertebrate fissure fauna and a new species of *Clevosaurus* (Lepidosauria: Rhynchocephalia) from Woodleaze Quarry, Gloucestershire, UK. *Proc. Geol. Assoc.* **126**, 402–416 (2015).

Langer, L., Ramezani, J., Da Rosa, A.S. U-Pb age constraints on dinosaur rise from south Brazil. *Gondwana Res.* **57**, 133-140 (2018).

Martínez RN, Apaldetti C, Colombi CE, Praderio A, Fernandez E, Malnis PS, Correa GA, Abelin D, Alcober O. A new sphenodontian (Lepidosauria: Rhynchocephalia) from the Late Triassic of Argentina and the early origin of the herbivore opisthodontians. *Proc. R. Soc. B.* **280**: 20132057 (2013)

Morton, J.D., Whitside D.I., Hethke, M., Benton. M.J. Biostratigraphy and geometric morphometrics of conchostracans (Crustacea, Branchiopoda) from the Late Triassic fissure deposits of Cromhall Quarry, UK. *Palaeontology* **60**,349-374 (2017).

Prasad, G.V.R. and Manhas, B.K., 2007. A New Docodont Mammal from the Jurassic Kota Formation of India. *Palaeontologia Electronica* **10**, 10.2.7A, 1-11 (2007).

Rauhut O.W.M., Heyng A.M., López-Arbarello A., Hecker A. A New Rhynchocephalian from the Late Jurassic of Germany with a dentition that is unique amongst Tetrapods. *PLoS One*, **7**, 1–9 (2012).

Reynoso, V.H. A "beaded" sphenodontian (Diapsida: Lepidosauria) From The Early Cretaceous Of Central Mexico. *Journal of Vertebrate Palaeontology* **17**, 52-59 (1997).

Reynoso, V.H. An Unusual Aquatic Sphenodontian (Reptilia: Diapsida) From The Tlayua Formation (Albian), Central Mexico. *J. Paleont.* **74**, 133-148 (2000).

Reynoso, V.H., J. M. Clark. A dwarf sphenodontian from the Huizachal Formation of Tamaulipas, Mexico. *Journal of Vertebrate Paleontology*, 18:333–339 (1998).

Saila, L.K. A new species of the sphenodontian reptile *Clevosaurus* from the Lower Jurassic of South Wales. *Palaeontology* **48**, 817-832 (2005).

T. E. Rasmussen and George Callison. A New Herbivorous Sphenodontid (Rhynchocephalia: Reptilia) from the Jurassic of Colorado. *Journal of Paleontology* **55**, 1109-1116 (1981).

Tanner, L.H. Formal definition of the Lower Jurassic McCoy Brook Formation, Fundy Rift Basin, eastern Canada. *Atlantic Geology* **32**,127-135 (1996).

Throckmorton, G.S., Hopson, J.A., Parks, P. A Redescription Of Toxolophosaurus Cloudi Olson, A Lower Cretaceous Herbivorous Sphenodontid Reptile. *Journal of Paleontology* **55**, 586-597 (1981).

Vijaya, Prasad, G.V.R. Age of the Kota Formation, Pranhita-Godavari Valley, India: a palynological approach. *Journal of the Palaeontological Society of India* **46**,77-93. (2001).

Whiteside, D.I. The head skeleton of the Rhaetian sphenodontid *Diphydontosaurus avonis* gen. et. sp. nov. and the modernizing of a living fossil. *Philosophical Transactions of the Royal Society, London B* **312**, 379–430 (1986).

Whiteside, D.I., Duffin, C.J., Gill, P.G., Marshall, J.E.A., and Benton, M.J. The Late Triassic and Early Jurassic fissure faunas from Bristol and South Wales: Stratigraphy and setting. *Palaeontologia Polonica* **67**, 257–287 (2017).

Worthy, T. H., Tennyson, A. J. D., Archer, M., Musser, A. M., Hand, S. J., Jones, C., Douglas, B. J., McNamara, J. A. & Beck, R. M. D. Miocene mammal reveals a Mesozoic

ghost lineage on insular New Zealand, southwest Pacific. *Proc. Natl Acad. Sci.* **103**, 19 419–19 423 (2006).

Worthy, T. H., Tennyson, A. J. D., Jones, C., McNamara, J. A. & Douglas, B. J. Miocene waterfowl and other birds from central Otago, New Zealand. *J. Syst. Palaeontol.* **5**, 1–39 (2007).

Zerfass, H. et al. Sequence stratigraphy of continental Triassic strata of Southernmost Brazil: A contribution to Southwestern Gondwana palaeogeography and palaeoclimate. *Sediment. Geol.* **161**, 85–105 (2003).
